# Restorative Management of Molar Incisor Hypomineralization–Affected Molars With Lithium Disilicate Overlays: An 8‐Year Follow‐Up Case Report

**DOI:** 10.1155/crid/1190624

**Published:** 2026-08-31

**Authors:** Ibtissem Grira, Bouthaina Mahjoubi, Nabiha Douki

**Affiliations:** ^1^ Department of Dental Medicine, Sahloul Hospital of Sousse, Faculty of Dental Medicine, Laboratory of Research in Oral Health and Maxillo-Facial Rehabilitation (LR12ES11), University of Monastir, Monastir, Tunisia, um.rnu.tn; ^2^ Department of Dental Medicine, Moknine Hospital, Faculty of Dental Medicine, University of Monastir, Monastir, Tunisia, um.rnu.tn

**Keywords:** adhesive dentistry, case report, ceramic overlays, indirect restorations, molar incisor hypomineralization (MIH), tooth preservation

## Abstract

Molar incisor hypomineralization (MIH) is a qualitative enamel defect characterized by reduced mineralization, increased porosity, and compromised mechanical properties, making affected teeth highly susceptible to posteruptive breakdown, hypersensitivity, and restorative failure. Conventional direct restorations often exhibit limited longevity in severely affected molars due to weak enamel bonding and structural fragility. Consequently, indirect restorations with cuspal coverage have been increasingly recommended as a more durable and protective treatment option. This clinical case reports the restorative management of MIH‐affected maxillary first molars in a 24‐year‐old patient using lithium disilicate ceramic overlays. Following the removal of defective amalgam restorations, a minimally invasive tooth preparation with a butt‐joint design was performed, combined with immediate dentin sealing to enhance adhesion. Impressions were taken using polyvinylsiloxane materials, and overlays were fabricated via CAD/CAM technology. Adhesive cementation was carried out under strict isolation, including surface treatment of both the restoration and dental tissues. The use of lithium disilicate overlays provided a conservative yet effective approach, ensuring reinforcement of weakened tooth structure while preserving remaining sound tissues. The literature supports the high survival rates of such restorations in MIH cases, with reduced hypersensitivity and satisfactory functional and aesthetic outcomes. In conclusion, lithium disilicate overlays represent a reliable and minimally invasive solution for the rehabilitation of compromised MIH molars. Careful case selection, proper adhesive protocols, and regular follow‐up are essential to ensure long‐term success, particularly given the inherent fragility of MIH‐affected enamel.

## 1. Introduction

Molar incisor hypomineralization (MIH) is a qualitative enamel defect caused by disturbed amelogenesis; the enamel is of normal thickness but has increased porosity, reduced hardness, and higher organic content, making it prone to posteruptive breakdown, caries, and hypersensitivity [1, 2].

Clinically, MIH most often affects first permanent molars and incisors and can compromise function, esthetics, and oral health–related quality of life in children and adolescents [2, 3].

Because hypomineralized enamel bonds less predictably and fractures easily under masticatory load, conventional direct restorations often show higher failure and marginal breakdown in severely affected molars [3, 4].

Systematic reviews and guidelines, therefore, recommend indirect, cuspal coverage restorations (inlays/onlays/overlays or partial crowns) for moderate–severe MIH once cooperation and eruption stage allow, as these provide improved protection of weakened cusps and better long‐term survival (0%–7% annual failure) compared with many direct materials [1, 3–5].

Ceramic overlays—especially lithium disilicate, zirconia‐reinforced glass‐ceramic, and CAD/CAM‐fabricated ceramics—combine high flexural strength, wear resistance, and esthetics and are considered a gold‐standard option when margins can be placed in sound enamel and adhesive cementation is feasible [1, 2, 6].

Recent randomized and cohort studies in MIH molars report high short‐ to medium‐term survival for CAD/CAM ceramic overlays in children, with marked reductions in hypersensitivity and satisfactory anatomy and function [2, 6, 7].

This clinical case describes the restorative management of MIH‐affected permanent molars using bonded ceramic overlays as a minimally invasive, definitive solution aimed at restoring function, relieving hypersensitivity, and preserving tooth structure.

## 2. Clinical Report

**Figure 1:**
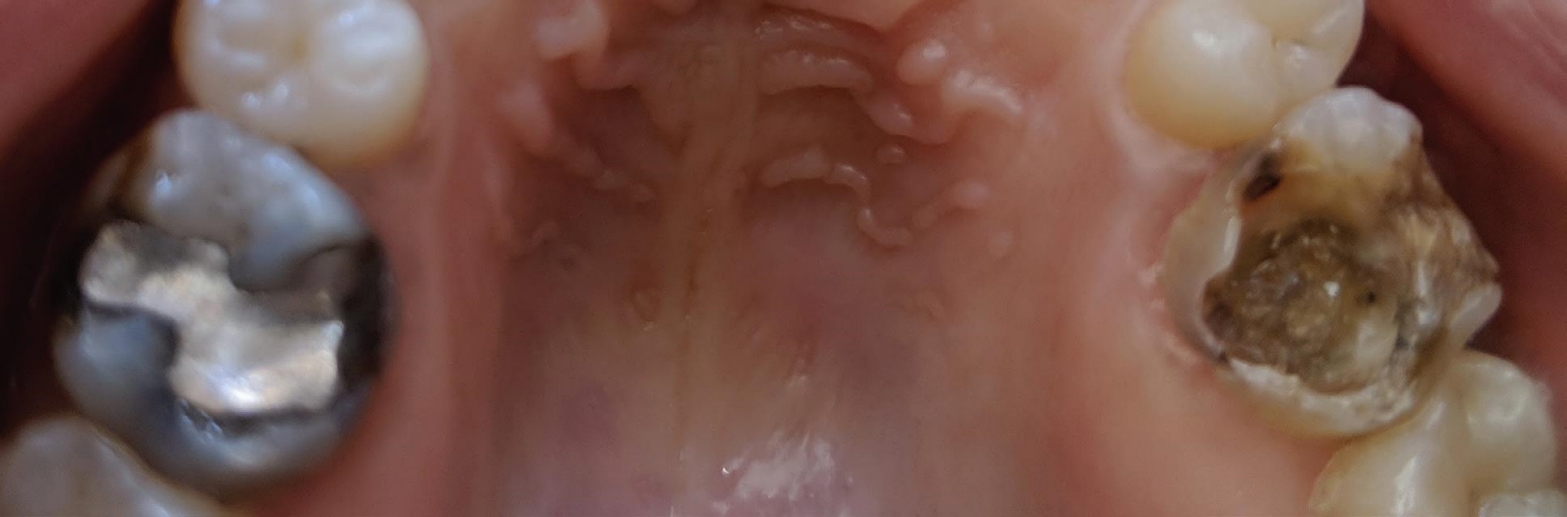
Preoperative occlusal view of MIH‐affected molars.

A 24‐year‐old female patient, in good general health, consulted the dental department at Sahloul hospital in June 2018 to restore her two first upper molars restored with amalgam since the age of 10 years. This case report focused on the restoration of both of the maxillary molars. Written informed consent was obtained from the patient for publication of the clinical information and accompanying clinical and radiographic images. On physical examination, the patient exhibited good oral health overall; however, she was aesthetically affected by abnormalities in the first molars. Inspection revealed the presence of a healthy periodontium and vital teeth. Based on the clinical examination, a diagnosis of MIH was made. Mild demarcated opacities were also observed on the maxillary central incisors without structural breakdown. The patient did not report spontaneous pain or discomfort during mastication. Clinical examination was used to determine the extent of structural enamel defects and to establish the treatment plan. The retro‐alveolar radiograph showed the absence of periapical complications. The patient expressed her need for an aesthetic and minimally invasive restoration of her teeth (Figure 1). The affected molars had never received definitive indirect restorations before the present treatment. After a global clinical examination, ceramic overlays, partial indirect restorations covering all cusps [8], were decided to enable the conservation of the remaining dental structure and also help in the reinforcement of a compromised tooth.

### 2.1. Tooth Preparation Design

**Figure 2:**
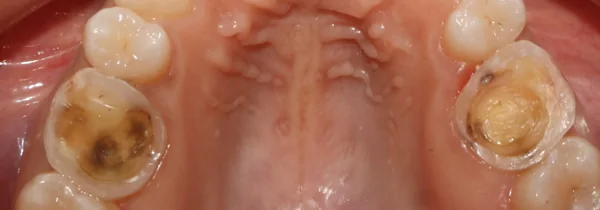
Occlusal view of the overlay preparation design.

**Figure 3:**
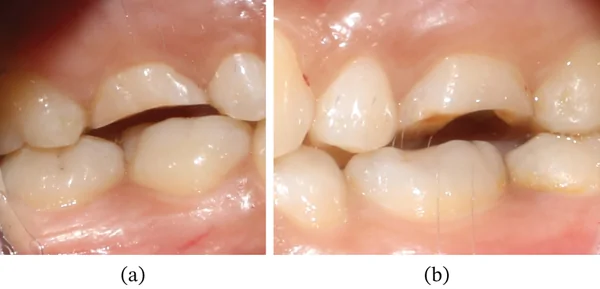
(a,b) Buccal view of the prosthetic space.

The clinical procedures involved, at first, the elimination of the amalgam restoration with a tungsten cylindrical bur. After that, the teeth were prepared for a butt‐joint design (BJD), which is useful for less invasive indirect occlusal restorations. A flat‐end diamond bur was used to prepare the occlusal box with rounded internal angles and an 8° convergence angle. A depth‐cut bur was then used to prepare depth‐oriented occlusal grooves, followed by an occlusal reduction of 1.5 mm, using a diamond wheel bur (Figures 2 and 3).

### 2.2. Immediate Dentin Sealing (IDS)

An IDS was performed directly after teeth preparation. It involved dentin etching with 37 of phosphoric acid for 15 s (Dentoetch, 37% phosphoric acid gel, 5 mL; C), followed by 1 min of rinsing and soft drying to leave the surface visibly moist. A universal adhesive (Meta P&Bond, MetaBiomed, 5 g) was applied and light‐cured for 20 s with a light‐curing device (B‐Woodpecker Cordless LED Curing Light, 1000 mW/cm^2^).

### 2.3. Impression Taking

A one‐step impression technique was used with high‐ and low‐viscosity addition polyvinylsiloxane materials (Elite HD+, Putty Soft and Light Body; Zhermack, Dentsply Sirona, Italy). Bis‐acryl interim restorations were fabricated (DentoCrown HD, Itena, France).

### 2.4. Overlay Fabrication

**Figure 4:**
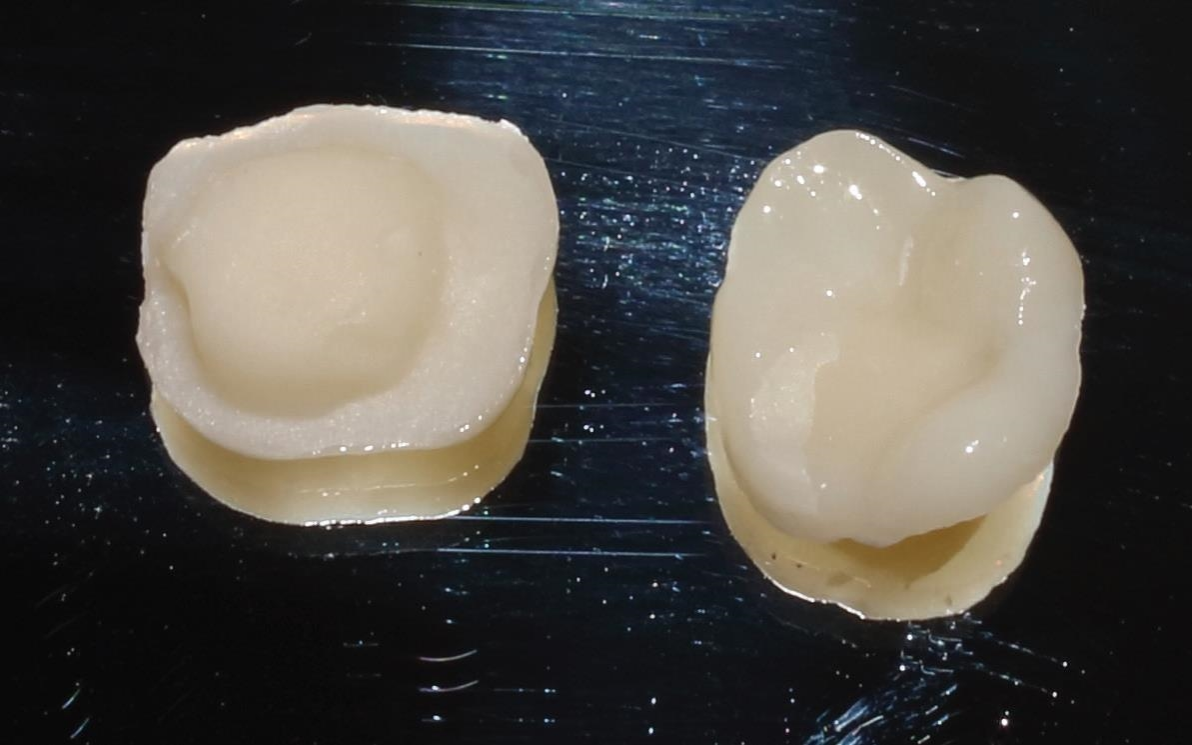
Lithium disilicate overlays.

Overlays were designed by Exocad software and were milled using lithium disilicate for CEREC and inLab (Ivoclar Vivadent, Schaan, Liechtenstein) (Figure 4).

### 2.5. Bonding Protocol

**Figure 5:**
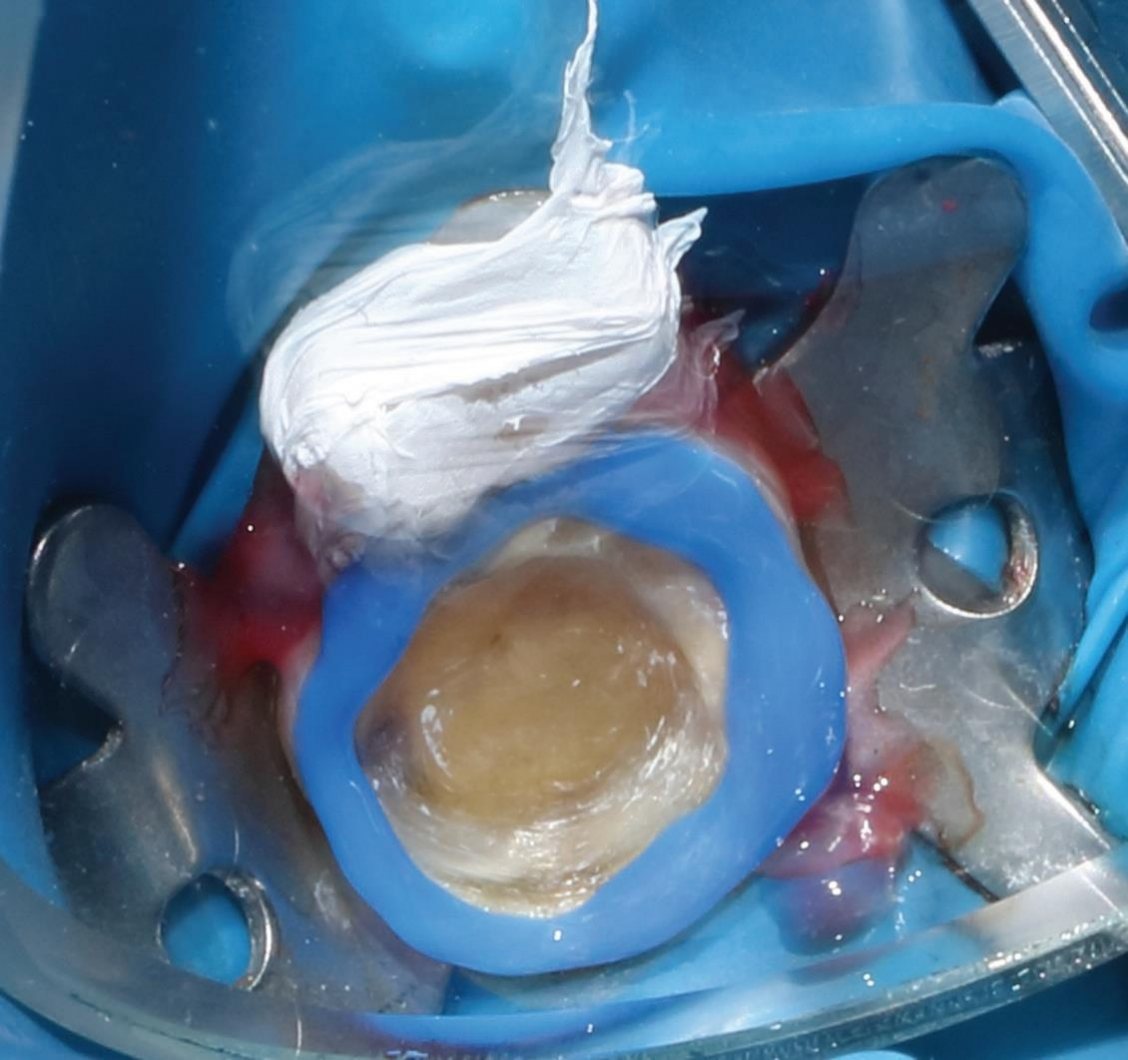
Selective etching of the enamel for 30 s.

Absolute isolation was performed using clamps and rubber dam. Since the overlay restorations were made from lithium disilicate, the bonding protocol consisted of the treatment of the intaglio surface of the overlays and the treatment of dental tissues. The intaglio was etched using 5% hydrofluoric acid (porcelain etch 5%, Ultradent, France) for 20 s, followed by rinsing and air‐drying. A universal primer (Monobond Plus, Universal Primer, Ivoclar Vivadent, Schaan, Liechtenstein) was then applied for 60 s and air‐dried. As the IDS was previously performed, only enamel was etched using 37% phosphoric acid for 30 s [9], followed by rinsing and air‐drying (Figure 5). To improve bond strength, the application of NAOCL was made after that.

In fact, etching refines enamel porosity, facilitating protein degradation by NaOCl and making the enamel crystals more accessible to the adhesive [9].

A three‐step etch‐and‐rinse adhesive (Syntac, light‐curing total‐etch adhesive, Ivoclar Vivadent) was applied following the manufacturer′s instructions.

**Figure 6:**
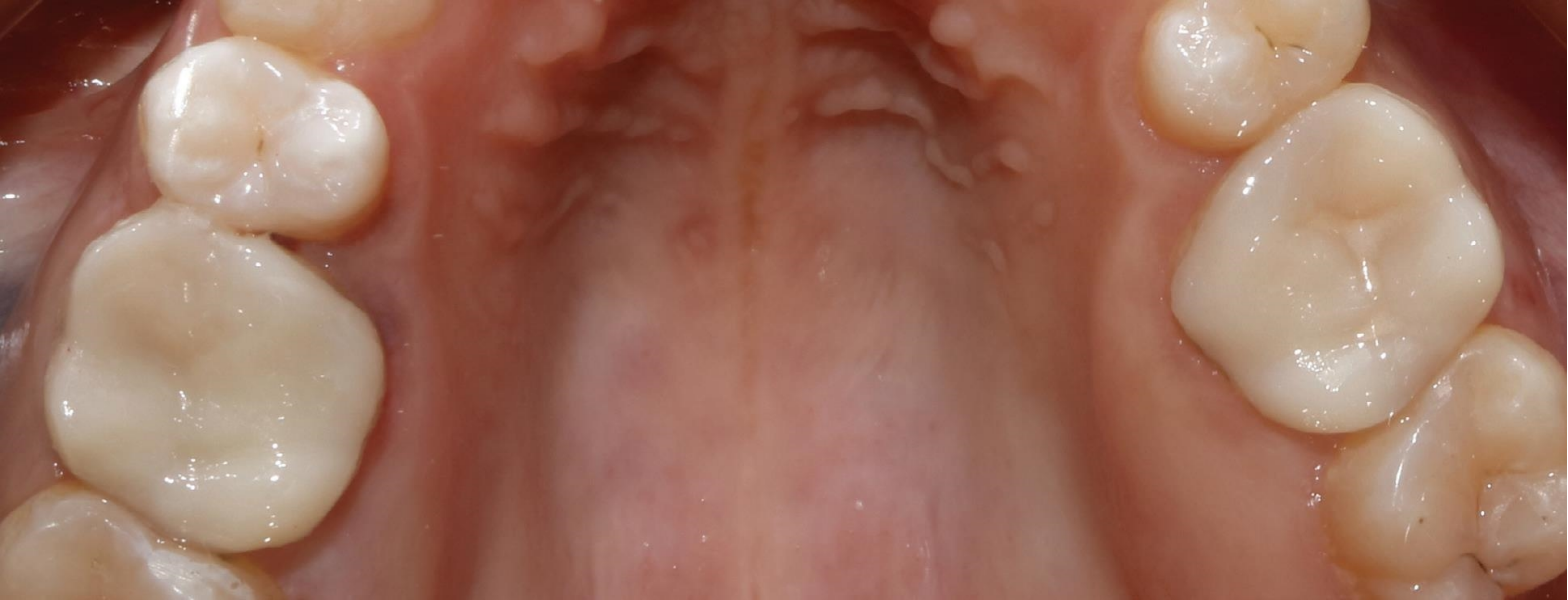
Final occlusal result after bonding of Lithium Disilicate overlays.

**Figure 7:**
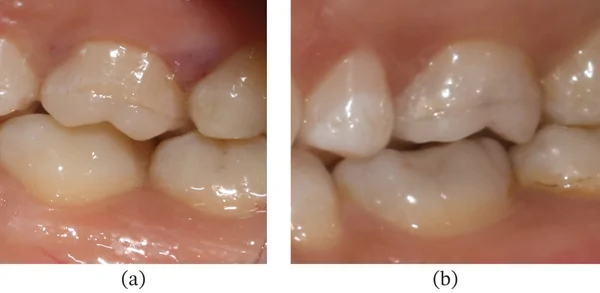
Buccal view after bonding showing good marginal adaptation and esthetic integration: (a) right upper first molar (16) and (b) left upper first molar (26).

Overlays were bonded with a dual‐cure and light‐cure luting composite (Variolink N, Professional Set, Ivoclar Vivadent). Excess cement was initially removed after a 3‐s flash light polymerization. Finally, 40 s of light curing was performed on each surface under a layer of glycerine gel, followed by finishing using polishing wheels (Figures 6 and 7). The patient reported complete satisfaction with both the aesthetic appearance and masticatory function of the restoration.

### 2.6. Follow‐Ups

The patient was contacted by telephone and invited to attend a follow‐up examination, scheduled 1, 3, and 8 years after the bonding session. Restorations were evaluated independently by two examiners according to the FDI World Dental Federation clinical criteria [10] for direct and indirect restorations. Prior to the assessment, both examiners underwent calibration to ensure consistency in applying the evaluation criteria, achieving an interexaminer agreement of over 90%.

**Figure 8:**
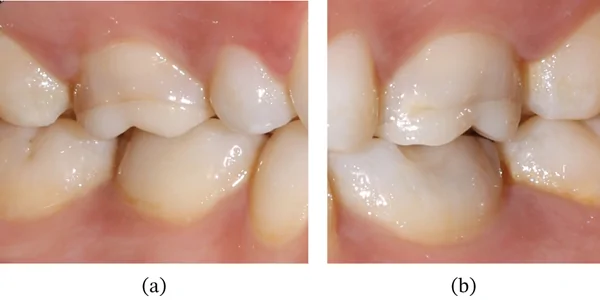
(a,b) Buccal view at 8‐year follow‐up of the overlay restorations.

**Figure 9:**
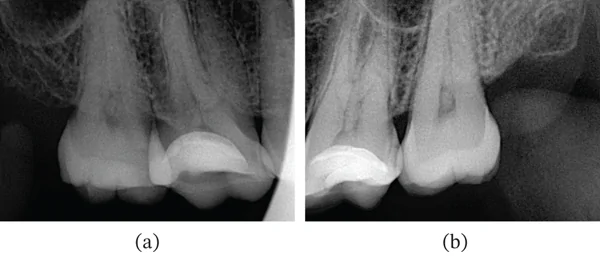
Periapical radiograph showing MIH‐affected molars with overlay restorations on (a) 16 and (b) 26.

The assessed parameters included fracture and retention of the restorative material, the presence of tooth cracks or fractures, and recurrence of the initial pathology. As a result, no failures were observed. Accordingly, 1‐, 3‐, and 8‐year survival was 100%. At the 8‐year recall, the lithium disilicate overlays demonstrated excellent marginal adaptation, color stability, and preservation of the remaining tooth structure, highlighting the potential longevity of this minimally invasive restorative approach in MIH‐affected molars (Figures 8 and 9). Achieving optimal aesthetic integration in MIH‐affected teeth remains challenging because the surrounding enamel often exhibits variations in opacity, color, and mineralization. Consequently, a slight transition between the restoration and the adjacent tooth structure may remain visible despite meticulous restorative procedures. In the present case, finishing and polishing procedures were performed after adhesive cementation, and the restorations were periodically monitored during follow‐up. The patient remained satisfied with both the functional and aesthetic outcomes over the 8‐year observation period.

## 3. Discussion

MIH creates porous, protein‐rich enamel with reduced hardness, which makes adhesive procedures less predictable and restorations more failure‐prone. Compared with sound enamel, MIH‐affected enamel exhibits compromised structural and mechanical properties, including reduced mineral density, increased protein content, greater porosity, and lower hardness and elastic modulus. These alterations are accompanied by disorganized enamel crystals with enlarged prism sheaths, resulting in a weakened substrate and less predictable adhesive bonding. In contrast, bonding to the underlying dentin is generally considered less challenging and is not regarded as the primary limiting factor [9, 11–13].

These features produce shallow or irregular etching patterns and a porous, fracture‐prone resin–enamel interface, leading to cohesive failures in enamel rather than purely adhesive failures. Bonding strategies must adapt to the altered substrate and often to severe hypersensitivity in young patients [9, 11, 14].

In contrast, MIH‐affected dentin can generally be bonded conventionally; microtensile/shear bond strengths in dentin are comparable with sound dentin and show few pretest failures [11, 15].

Multiple in vitro studies show significantly lower bond strength to MIH enamel than to sound enamel, for both etch‐and‐rinse and self‐etch systems [9, 11, 12, 15].

Pretest failures and cohesive fractures in the weak MIH enamel are frequent, indicating that enamel quality, not the adhesive per se, is often the main weak link [9, 11, 15]When possible, place restoration margins on clinically sound enamel; this remains a core recommendation to improve longevity [3, 13].

An “invasive” approach removing all hypomineralized enamel to sound tissue improved survival and yielded bond similar to sound enamel in one study; however, this sacrifices more tooth structure. Conservative approaches must accept a weaker substrate and possible chipping around margins [9].

Bonding to MIH‐affected teeth is mainly compromised at the enamel level. Dentin bonding is usually acceptable, but weak, porous MIH enamel reduces bond strength and promotes cohesive failures. Current evidence supports careful substrate assessment, preference for margins on sound enamel, possible use of etch‐and‐rinse plus optional deproteinization or resin infiltration, and emerging interest in bioactive glass–containing adhesives to enhance durability [11, 14].

In severe MIH, indirect restorations are often preferred once eruption is complete and cooperation allows, because they bypass weak enamel and provide long‐term protection. Several ceramic options (and designs) have now been tested specifically in MIH molars. CAD/CAM ceramic overlays/onlays (zirconia, e.max CAD) on MIH FPMs showed all restorations functional at 12 months, with only a few fractures or debonds [6].

Occlusal veneers and endocrowns made from Vita Suprinity (zirconia‐reinforced lithium silicate) and Vita Enamic (hybrid ceramic) had similarly high success over 18 months; scoping and systematic reviews confirm both as reliable partial‐coverage options in severe MIH [1, 2, 16].

For indirect restoration of MIH‐affected teeth, the best‐supported options are bonded lithium disilicate or zirconia‐reinforced glass‐ceramics and hybrid/resin‐matrix ceramics for stress‐absorbing partial coverage in young molars; zirconia overlays are an alternative where maximum strength is required.

Resin‐matrix/hybrid ceramics (e.g., Vita Enamic and CAD/CAM nanohybrid composites) offer easier repair and shock absorption with MIH‐compromised enamel [2, 17].

However, lithium disilicate or zirconia‐reinforced lithium silicate overlays/onlays/endocrowns are well supported for partial coverage in MIH and in worn dentition generally [6, 18].

For MIH molars restored with overlays/onlays, follow‐up is important because the underlying enamel is fragile and patients are often young. Clinical studies on indirect overlays in MIH report reviews from a few months up to 3–6 years.

A retrospective cohort of CAD/CAM ceramics in severe MIH followed patients for about 3 years, showing 100% survival at 36 months [19].

Systematic reviews of indirect restorations in MIH include follow‐ups from 2 months to 6 years, with generally very low failure rates (0%–7% annual failure) [1, 5].

Systematic and umbrella reviews highlight that indirect restorations in severe MIH have the best long‐term survival among restorative options, but evidence is still limited and heterogeneous, so regular clinical and radiographic review is recommended rather than assuming lifelong permanence [1, 3, 5, 19].

Across clinical trials and reviews, the key follow‐up parameters for MIH overlays include retention and debonding—particularly noted in zirconia overlays, with reports of debonding events within the first year [6]—alongside fracture or loss of anatomic form, with occasional failures observed in certain ceramic or composite materials [7]. Marginal adaptation and the occurrence of secondary caries, especially at cervical margins in sound enamel, are also critical considerations [5, 20].

In addition, pulpal and periodontal status, as well as hypersensitivity, are closely monitored, with most studies reporting complete resolution of pre‐existing sensitivity following onlay or overlay placement [3, 6]. Finally, occlusion and craniofacial growth remain essential parameters in children and adolescents, given the ongoing processes of tooth eruption and jaw development [19, 21].

## 4. Summary

MIH is a qualitative enamel defect characterized by reduced mineralization, increased porosity, and poor mechanical properties, which make affected teeth prone to posteruptive breakdown, hypersensitivity, and restorative failure. Due to the compromised bonding capacity of hypomineralized enamel, conventional direct restorations often show limited longevity, especially in severely affected molars. As a result, indirect restorations with cuspal coverage are increasingly recommended for improved durability and protection.

This article presents an 8‐year clinical case involving a 24‐year‐old patient treated for MIH‐affected maxillary first molars using lithium disilicate ceramic overlays. After removal of defective amalgam restorations, a minimally invasive tooth preparation with a BJD was performed, combined with IDS to enhance adhesion. CAD/CAM technology was used to fabricate the overlays, which were adhesively bonded under strict isolation following appropriate surface treatments.

The clinical outcomes were highly successful, with no failures reported over 1‐, 3‐, and 8‐year follow‐ups, resulting in a 100% survival rate. The restorations demonstrated excellent functional, aesthetic, and biological integration, along with resolution of hypersensitivity.

The patient expressed satisfaction with the treatment and the final outcome. She was pleased with the natural appearance and comfort of the restorations and remained satisfied with their function and aesthetics throughout the follow‐up period.

The discussion highlights the challenges of bonding to MIH‐affected enamel due to its structural weakness and emphasizes the importance of placing restoration margins on sound enamel when possible. Current evidence supports the use of indirect ceramic restorations, particularly lithium disilicate, as a reliable and minimally invasive solution for managing moderate to severe MIH cases.

In conclusion, lithium disilicate overlays represent an effective long‐term treatment option for MIH‐affected molars, provided that appropriate adhesive protocols, careful case selection, and regular follow‐up are ensured.

## Funding

No funding was received for this manuscript.

## Disclosure

All authors have read and approved the final version of the manuscript. Dr Ibtissem Grira had full access to all of the data in this study and takes complete responsibility for the integrity of the data and the accuracy of the data analysis.

## Conflicts of Interest

The authors declare no conflicts of interest.

## Data Availability

The authors confirm that the data supporting the findings of this study are available within the article and/or its supporting information.
